# An obese patient with acute cholecystitis, nonalcoholic steatohepatitis and cirrhosis: A case report

**DOI:** 10.1016/j.ijscr.2020.01.028

**Published:** 2020-01-27

**Authors:** Toru Zuiki, Jun Ohki, Toshio Horiuchi, Alan Kawarai Lefor, Fuyumi Shirakata, Yuka Hirota, Norio Hirota

**Affiliations:** a Department of Surgery, Yuki Hospital, Yuki 9629-1, Yuki City, Ibaraki, Japan; b Department of Surgery, Jichi Medical University, Yakushiji 3311-1, Shimotsuke City, Tochigi, Japan; c Hirota Institute for Surgical Pathology, Idaimae 3-6-5, Shimotuke City, Tochigi, Japan; d Department of Human Pathology, Jichi Medical University, Yakushiji 3311-1, Shimotsuke City, Tochigi, Japan

**Keywords:** NASH, nonalcoholic steatohepatitis, PTGBD, percutaneous transhepatic gallbladder drainage, BMI, body mass index, CT, computed tomography, NAFLD, non-alcoholic fatty liver disease, Laparoscopic cholecystostomy, Low-carbohydrate diet, Nonalcoholic steatohepatitis, Acute cholecystitis, Liver cirrhosis, Obesity

## Abstract

**Introduction:**

Laparoscopic cholecystectomy for patients with acute cholecystitis and liver cirrhosis is associated with increased risk. We present an obese patient with acute cholecystitis and liver cirrhosis caused by nonalcoholic steatohepatitis (NASH), who was successfully managed with laparoscopic cholecystostomy and a low-carbohydrate diet with exercise.

**Presentation of case:**

A 61-year-old woman presented with right upper quadrant abdominal pain. Ultrasonography and computed tomography were consistent with acute cholecystitis with multiple stones and cirrhosis. The patient had no history of alcohol intake, and serologic tests were negative. The patient’s body mass index was 39 kg/m^2^ (154 cm, 93 kg) and NASH was suspected. Percutaneous transhepatic drainage was impossible because of the anatomic position of the gallbladder. Emergency laparoscopic cholecystostomy was performed initially for drainage. A low-carbohydrate diet and exercise were started for weight loss and her weight reduced by 19 kg over three months. Open cholecystectomy was performed uneventfully, and liver biopsy suggested NASH.

**Discussion:**

Laparoscopic cholecystostomy is a reasonable temporary alternative to cholecystectomy in patients with acute cholecystitis and increased surgical risk. Weight loss with diet and exercise can be effective in patients with NASH. A low-carbohydrate diet is a reasonable treatment for NASH, because glucose is converted to triglycerides and stored as lipid in the liver.

**Conclusion:**

Laparoscopic cholecystostomy was effective in this obese patient with acute cholecystitis and NASH cirrhosis. Using a low-carbohydrate diet with exercise, her weight decreased, and subsequent open cholecystectomy was uneventful.

## Highlights

• Laparoscopic cholecystostomy is a safe alternative in high-risk acute cholecystitis.
• A low-carbohydrate diet with exercise is effective in nonalcoholic steatohepatitis.
• Liver fibrosis due to NASH cirrhosis improves with limited sugar intake.

## 1 Introduction

Urgent laparoscopic cholecystectomy is a standard treatment for low risk patients with acute cholecystitis. The advantages of laparoscopic cholecystectomy for selected patients with well compensated liver cirrhosis have been recognized [1,2]. However, the postoperative morbidity in these patients is higher than in patients without cirrhosis in those reports. In patients with decompensated cirrhosis, even a minimally invasive procedure may lead to life-threatening complications. Patients for whom cholecystectomy is associated with increased risk or percutaneous transhepatic gallbladder drainage (PTGBD) is difficult, may benefit from initial open cholecystostomy as a bridge to cholecystectomy. A laparoscopic cholecystostomy is even less invasive. Several reports have shown that a low-carbohydrate diet is effective in obese patients [[3], [4], [5]], and weight loss by diet and exercise are both effective in patients with nonalcoholic steatohepatitis (NASH) [[6], [7], [8], [9]]. We present an obese patient with acute cholecystitis and liver cirrhosis caused by NASH, who was successfully managed with laparoscopic cholecystostomy and low-carbohydrate diet with exercise, followed by open cholecystectomy. This work is reported in conformity with the SCARE criteria [10].

## 2 Presentation of case

**Fig. 1:**
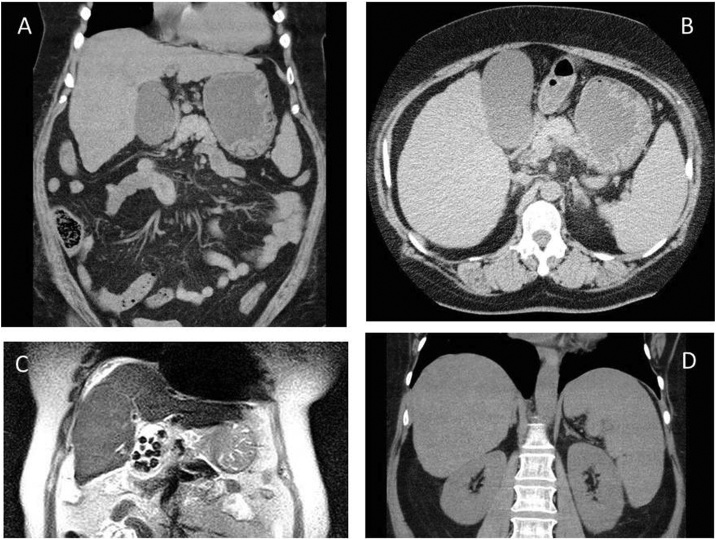
Computed tomography (CT) scan and magnetic resonance imaging findings. A. Irregular surface of the liver and dilatation of the gallbladder are seen. B. The gallbladder is medial. C. MRI revealed multiple gall stones. D. Splenomegaly is seen in the coronal plane.

A 61-year-old female presented with severe abdominal pain. Her medical history was unremarkable. There was no history of significant illness or previous abdominal surgery. The patient had no history of alcohol intake. The patient’s body mass index (BMI) was 39 kg/m^2^ (154 cm, 93 kg). Rebound tenderness and Murphy’s sign were present in the right upper quadrant. Ultrasonography and magnetic resonance imaging revealed gallbladder wall thickening to 6 mm with multiple stones consistent with acute cholecystitis. Computed tomography (CT) scan revealed an irregular liver surface and splenomegaly (Fig. 1). The gallbladder was somewhat medially located and dilated with a long axis of 104 mm and a short axis of 53 mm. Obvious collateral vessels in the abdominal cavity and ascites were not present. Laboratory data showed no abnormalities on admission, but the white blood cell count and serum C-reactive protein were elevated the following day and arterial blood gas analysis showed hypoxia (Table 1).

**Table 1:** Laboratory Data.

	Day 1	Day 2	
White Blood Cell count	5250	10650	/μl
Red Blood Cell count	388		×10^3^μl
Hemoglobin	10.7	10.9	g/dl
Hematocrit	32.6		%
MCV	84		fl
MCH	27.6		pg
MCHC	32.8		%
Platelet count	10.9	12	×10^4^μl

Prothrombin time		61	%
PT-INR		1.31	INR
Activated partial thromboplastin time		33	sec
Arterial blood gas analysis			
pH		7.497	
PO_2_		50	torr
PCO_2_		33.5	torr
HCO_3_^−^		25.7	mmol/l
Base Excess		2.5	mmol/l
SO_2_		87.9	%

Total Protein	7	6.6	g/dl
Albumin	4	3.6	g/dl
Asparate aminotransferase	44	32	IU/L
Alanine aminotransferase	37	31	IU/L
Alkaline phosphatase	396	339	IU/L
Lactate dehydrogenase	200	178	IU/L
Total bilirubin	1.72	2.34	mg/dl
Gamma glutamyl transpeptidase		84	IU/L
Cholinesterase		276	IU/L
Creatine phosphokinase		138	IU/L
Total cholesterol	180	162	mg/dl
Blood Urea Nitrogen	10.6	10.7	mg/dl
Creatinine	0.39	0.46	mg/dl
Sodium	141	136	mEq/L
Potassium	3.8	3.4	mEq/L
Chloride	105	103	mEq/L
Glucose	119	131	mg/dl
Hemoglobin A1c	5.5		%
C-reactive protein	0.23	4.5	mg/dl

MCV: erythrocyte mean corpuscular volume, MCH: erythrocyte mean corpuscular hemoglobin, MCHC: erythrocyte mean corpuscular hemoglobin concentration, PT-INR: prothrombin time international normalized ratio, pH: potential of hydrogen, PO_2_: oxygen partial pressure, PCO_2_: carbon dioxide partial pressure, HCO_3_^−^: carbonated hydrogen ion, SO_2_: oxygen saturation.

**Fig. 2:**
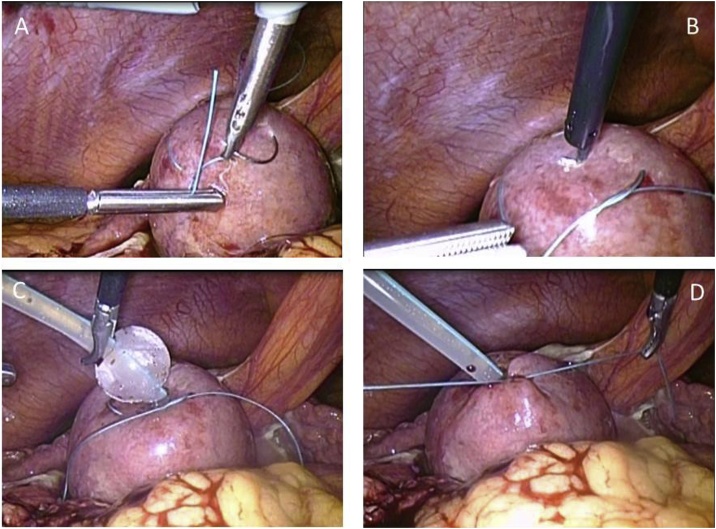
Laparoscopic cholecystostomy. A. A purse-string suture was placed in the gallbladder before puncture. B. A small hole was made in the center of the purse-string suture. C. A balloon catheter was prepared. D. The purse-string suture was secured after insertion of a balloon catheter.

The patient’s abdominal pain was not relieved by treatment with antibiotics, and a fever to 39.5 °C developed. PTGBD was considered difficult because of the location of the gallbladder and there was no adequate window for a safe procedure. The Charlson comorbidity index [11] with age adjustment was 6 (moderate or severe liver disease: 3, age 61: +3). The American Society of Anesthesiologists physical status classification [12] was 3E. The severity grading was grade III (severe) acute cholecystitis (with respiratory dysfunction) according to the Tokyo guidelines 2018 [13]. Based on this assessment, emergency laparoscopic or open cholecystectomy was thought to be associated with excessive risk [14]. Laparoscopic cholecystostomy was performed under general anesthesia on the third hospital day to establish drainage. A 12-mm umbilical trocar was placed through a small incision, and three 5-mm working ports were placed in the right lateral, right subcostal, and left medial abdomen. Omental adhesions were peeled from the fundus of the gallbladder and a purse-string suture [15] was placed in the fundus. A 16 F balloon catheter was inserted through the right subcostal trocar and then placed into the gallbladder through the purse-string suture which was secured after the balloon was inflated (operative time: 48 min, estimated blood loss: minimal) (Fig. 2). Antibiotic therapy (Cefoperazone sodium and Sulbactum sodium) was given for five days and the patient became afebrile on the second postoperative day.

Further laboratory evaluation showed that the serum hepatitis B virus antigen, hepatitis C virus antibody, and anti-mitochondrial antibody were negative. NASH was considered to be the cause of cirrhosis. The plan at this point was to perform a delayed cholecystectomy.

**Fig. 3:**
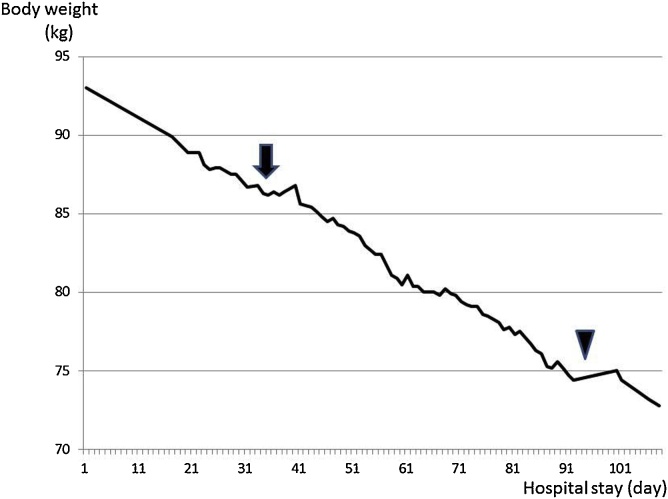
Change in weight. A low-carbohydrate diet with exercise was started on hospital day 35 (arrow). The patient’s weight reduced linearly, and cholecystectomy was performed on hospital day 93 (arrow head).

We recommended discharge with the cholecystostomy in place, but the patient and family strongly wanted to continue hospitalization until the cholecystectomy. During this time, a low-carbohydrate diet (70 g/day carbohydrates) and aerobic exercise more than thirty minutes daily were prescribed for weight loss before the cholecystectomy. A nutritionist and a physical therapist were actively involved, and the patient’s weight decreased by 19 kg (74 kg, BMI: 31 kg/m^2^) over three months (Fig. 3).

**Fig. 4:**
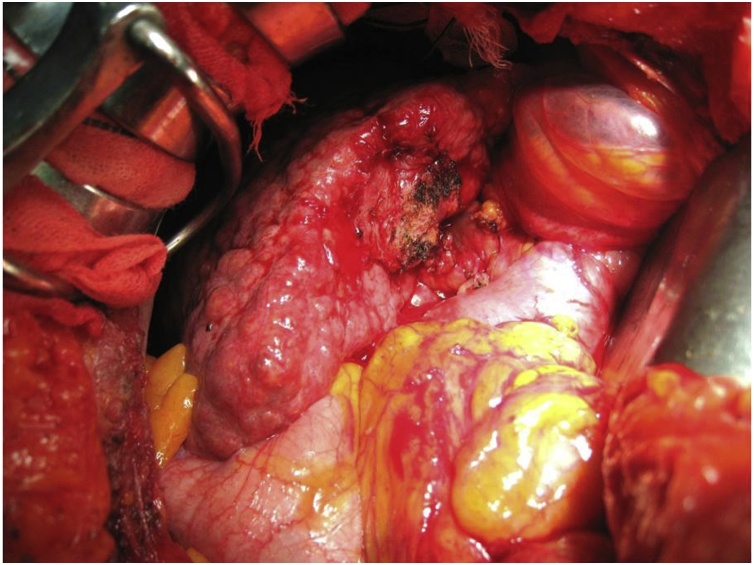
Intraoperative findings. After removing the anterior wall, the posterior wall of the gallbladder remaining on the liver bed was cauterized. The surface of the liver is irregular suggesting cirrhosis, and liver biopsy performed during the cholecystectomy.

**Fig. 5:**
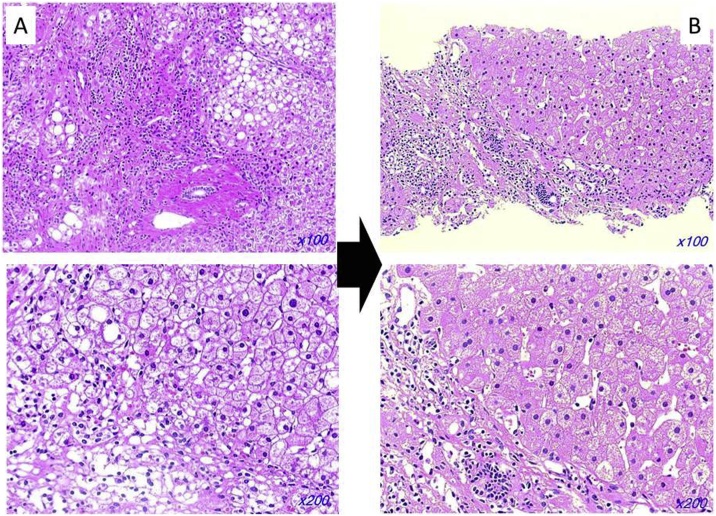
Pathological findings of liver biopsy specimen. A. Liver biopsy specimen revealed fatty changes with fibrosis and ballooning of hepatocytes, accompanying inflammatory cells around portal vein. B. Repeat biopsy one year after weight loss showed improvement of fibrosis and steatosis.

The operative findings at the first operation predicted that laparoscopic cholecystectomy would be difficult with the expectation of significant inflammation around the gallbladder. Open subtotal cholecystectomy was performed on hospital day 93. The gallbladder had multiple dense adhesions on the serosal surface as expected and was partially buried in the cirrhotic liver. The gallbladder wall was incised at the fundus of gallbladder, and the cholecystostomy tube and gallstones were removed. After removing the anterior wall, the posterior wall of the gallbladder remaining on the liver bed was cauterized. The cystic duct was ligated and divided (operative time: 71 min, estimated blood loss: 130 ml) (Fig. 4). The postoperative course was uneventful except for a minor wound infection which was treated with open wound care. The patient was discharged on postoperative day 20 after the subtotal cholecystectomy. Liver biopsy performed during the operative procedure revealed fatty changes with fibrosis and ballooning of hepatocytes, accompanying inflammatory cells around the portal vein and few Mallory-Denk bodies. The non-alcoholic fatty liver disease (NAFLD) activity score was 6, Brunt’s NASH grading was grade 2 (stage 4), and Matteoni’s classification was type 4 (Fig. 5A).

The patient continued a low-carbohydrate diet at home after discharge and received regular counseling by a nutritionist at the outpatient clinic. Repeat liver biopsy performed one year later showed improvement of fibrosis and steatosis. Repeat biopsy had an NAFLD activity score of 3, Brunt’s NASH grade 1 (stage 1a), and Matteoni’s classification of type 3 (Fig. 5B).

## 3 Discussion

The treatment strategy for this patient with acute cholecystitis is generally consistent with the Tokyo guidelines 2018 [13,14]. If the patient can tolerate surgery after assessment using the Charlson comorbidity index and American Society of Anesthesiologists physical status, early laparoscopic cholecystectomy is performed. If the patient is not a good candidate for early cholecystectomy, a less invasive treatment such as PTGBD is performed initially and delayed cholecystectomy is planned. PTGBD is sometimes selected as first a less invasive management strategy based on severity of illness in some elderly patients treated in our facility, and delayed open or laparoscopic cholecystectomy then performed. As stated in the guidelines, there is no evidence for the optimal timing of surgery after PTGBD and this should be determined on an individual basis [14]. Based on the guidelines and our experiences, we feel it is reasonable to wait 4–6 weeks until the inflammation around the gallbladder is resolved. Some elderly patients with drainage tubes in place are treated in our long-term care facility before cholecystectomy. Patients with acute cholecystitis with significant comorbidities such as decompensated liver cirrhosis or being anticoagulated are at increased risk even for PTGBD. Endoscopic trans-papillary gallbladder drainage [16] and endoscopic ultrasound-guided gallbladder drainage [17] have been recently described as alternatives, but they depend on the anatomy of the biliary-tract and are advanced endoscopic techniques requiring specialized training. Emergency cholecystectomy in such high-risk patients may lead to fatal postoperative complications. In the Tokyo Guidelines 2018, the evaluation of severity and treatment of patients with acute cholecystitis with liver cirrhosis is not explicitly stated [13]. The guidelines suggest that patients with cirrhosis are considered to have significant morbidity and should be treated carefully [14]. We have performed open cholecystostomy through a small subcostal incision using a balloon catheter in some high-risk patients and for patients without an adequate window for PTGBD, and obtained satisfactory results. It is a temporary and effective alternative in the patients with severe acute cholecystitis. Recently, we have performed this procedure laparoscopically and it may be less invasive for patients in less than ideal condition. Although we performed laparoscopic cholecystostomy under general anesthesia for the present patient after consultation with an anesthesiologist, it can be performed with local anesthesia and intravenous sedation. Laparoscopic cholecystostomy as an emergency procedure has been rarely reported [[18], [19], [20]], although it is reasonable for high-risk patients. The rarity of reports for this approach may be that laparoscopic cholecystectomy is recommended in guidelines and surgeons usually attempt this in low risk patients. In the Tokyo guidelines 2018, laparoscopic cholecystectomy for patients with severe acute cholecystitis is recommended to be performed only by expert laparoscopic surgeons at a specialized center with adequate intensive care facilities [14]. Laparoscopic cholecystostomy may be a good option for laparoscopic surgeons with less experience with such patients. A Delphi consensus cited in the Tokyo guidelines 2018 concluded that cholecystostomy is a rarely needed alternative to prevent bile duct injuries during laparoscopic cholecystectomy [21].

Several studies show that a low-carbohydrate diet is effective for weight loss in obese patients [[3], [4], [5]]. Weight loss including diet and exercise are effective in patients with NASH [[6], [7], [8], [9]]. NASH is exacerbated by sugar intake because glucose is converted to triglyceride and stored as lipids in the liver. Based on this metabolic mechanism, a low-carbohydrate diet is thought to be a reasonable treatment for patients with NASH. After therapy, liver fibrosis improved in the repeat liver biopsy specimen. Although fibrosis is often considered to be irreversible, this result shows that fibrosis in patients with NASH cirrhosis is reversible and improved by limitation of sugar intake [7]. Improvement in the pathological findings of steatosis after therapy can be expected to result in improved liver function in patients with NASH cirrhosis. Although we compared pathological findings only, the utility of gadoxetic acid enhanced MRI to evaluate liver function in cirrhosis has been reported [22]. It may be useful for minimally invasive evaluation of the therapeutic effects on NASH cirrhosis.

In theory, a low-carbohydrate diet is thought to be contraindicated in patients with liver cirrhosis, because gluconeogenesis is insufficient in the cirrhotic liver. However, patients with NASH cirrhosis are somewhat different, because a liver affected by NASH contains excess lipids as an energy supply through the synthesis of ketone bodies. In fact, this patient did not experience hypoglycemia or general fatigue despite consuming a low-carbohydrate diet and undergoing exercise therapy. However, eventually in the liver affected by NASH in which the lipids have disappeared and only fibrosis referred to as “burned-out NASH”, a low-carbohydrate diet may be contraindicated because of energy depletion. Further studies of a low-carbohydrate diet with exercise in patients with NASH cirrhosis are needed.

This treatment strategy may be poorly accepted by some patients, because it requires strong motivation. Adequate psychological and emotional support of obese patients in a weight loss program is essential. We believe that this patient accepted this therapy because of a limited time until the second operation, but this preoperative treatment was reasonable and minimally invasive. This patient wanted to continue hospitalization until second operation as many elderly Japanese patients readily accept long-term hospitalization, which may be different from the approach in other countries and cultures. This weight loss program could also be conducted in an outpatient clinic with a dedicated nutrition support team. This patient was able to maintain a low-carbohydrate diet and exercise at home until repeat liver biopsy one year after cholecystectomy, and her weight was maintained at 67 kg (BMI: 28 kg/m^2^). Dissemination of the benefits of a low-carbohydrate diet with exercise is needed for patients with NASH, and further physiological and pathological studies are needed.

## 4 Conclusion

Laparoscopic cholecystostomy was effective as the first step in the management of this obese patient with acute cholecystitis and NASH cirrhosis. Following a low-carbohydrate diet with exercise resulted in significant weight reduction, and cholecystectomy was safely performed. Fibrosis and steatosis caused by NASH cirrhosis improved after weight loss as shown by repeat liver biopsy.

## Sources of funding

This research did not receive any specific grant from funding agencies in the public, commercial, or not-for-profit sectors.

## Ethical approval

The need for ethical approval was waived in our institution for this study.

## Consent

Written informed consent was obtained from the patient for publication of this case report and accompanying images.

## Author contribution

TZ performed surgery, wrote the paper, made literature review, and drafted the manuscript. JO advised the management of this patient as an expert surgeon. TH and FS treated the patient and assisted surgery. NH and YH advised pathological findings as expert pathologists. AL reviewed the paper, and revised the manuscript.

## Registration of research studies

This is a case report and is not a clinical research, and not be registered in a publicly accessible database.

## Guarantor

The manuscript has been read and approved by all authors and is not under consideration for publication elsewhere. Dr. Ohki, Director of Yuki Hospital, is the Guarantor.

## Provenance and peer review

Not commissioned, externally peer-reviewed.

## Declaration of Competing Interest

All authors declare that they have no competing interests.
